# Effect of canonical NF-κB signaling pathway on the differentiation of rat dental epithelial stem cells

**DOI:** 10.1186/s13287-019-1252-7

**Published:** 2019-05-20

**Authors:** Yan Liang, Guoqing Chen, Yuzhi Yang, Ziyue Li, Tian Chen, Wenhua Sun, Mei Yu, Kuangwu Pan, Weihua Guo, Weidong Tian

**Affiliations:** 1 National Engineering Laboratory for Oral Regenerative Medicine, West China Hospital of Stomatology, Sichuan University, Chengdu, 610041 People’s Republic of China; 2 State Key Laboratory of Oral Diseases, West China Hospital of Stomatology, Sichuan University, Chengdu, 610041 People’s Republic of China; 3 Department of Oral and Maxillofacial Surgery, West China Hospital of Stomatology, Sichuan University, No.14, 3rd Section, Renmin South Road, Chengdu, 610041 People’s Republic of China; 4 Department of Head and Neck Oncology, West China Hospital of Stomatology, Sichuan University, Chengdu, 610041 People’s Republic of China; 5 Department of Pediatric Dentistry, West China College of Stomatology, Sichuan University, No.14, 3rd Section, Renmin South Road, Chengdu, 610041 People’s Republic of China

**Keywords:** Dental epithelial stem cell, Differentiation, NF-κB, Amelogenesis, TGF-β

## Abstract

**Background:**

Nuclear factor-κB (NF-κB), an important transcription factor, participates in many physiological and pathological processes such as growth, differentiation, organogenesis, apoptosis, inflammation, and immune response, including tooth development. However, it is still unknown whether NF-κB participates in the regulation of dental epithelial stem cells (DESCs) in postnatal rat incisors. Here, we investigated the specific differentiation regulatory mechanisms of the canonical NF-κB signaling pathway in DESCs and provided the mechanism of cross-talk involved in DESC differentiation.

**Methods:**

After adding the activator or inhibitor of the NF-κB signaling pathway, Western blot and quantitative real-time PCR were used to analyze the expressions of amelogenesis-related genes and proteins and canonical transforming growth factor-β (TGF-β) signaling. In addition, we used amelogenesis induction in vitro by adding the activator or inhibitor of the NF-κB signaling pathway to the amelogenesis-induction medium, respectively. Recombinant TGF-β was used to activate the TGF-β pathway, and SMAD7 siRNA was used to downregulate the expression of SMAD7 in DESCs.

**Results:**

We found that the expression of amelogenesis-related genes and proteins as well as TGF-β signaling were downregulated, while SMAD7 expression was increased in NF-κB-activated DESCs. In addition, NF-κB-inhibited DESCs exhibited opposite results compared with NF-κB-activated DESCs. Furthermore, the canonical NF-κB signaling pathway suppressed the canonical TGF-β-SMAD signaling by inducing SMAD7 expression involved in the regulation of DESC differentiation.

**Conclusions:**

These results indicate that the canonical NF-κB signaling pathway participated in the regulation of DESC differentiation, which was through upregulating SMAD7 expression and further suppressing the canonical TGF-β-SMAD signaling pathway.

**Electronic supplementary material:**

The online version of this article (10.1186/s13287-019-1252-7) contains supplementary material, which is available to authorized users.

## Background

Rodent incisors grow continuously throughout life, which relies on the dental epithelial cells residing in stem cell niches in the cervical loop [1, 2]. The cervical loop consists of the stellate reticulum, stratum intermedium, inner enamel epithelium, and outer enamel epithelium. The stellate reticulum, the central epithelial tissue of the cervical loop, acts as a stem cell reservoir [1]. The stem cells migrate from the stellate reticulum to the inner enamel epithelium and produce transit-amplifying cells. The transit-amplifying cells give rise to the pre-ameloblasts which further differentiate into ameloblasts forming enamel matrix. In contrast, the cervical loop disappeared and forms Hertwig epithelial root sheath which makes up the inner enamel epithelium and outer enamel epithelium and initiates root forming after crown forming in human teeth [2]. Hence, dental epithelial stem cells (DESCs) played a significant role in tooth development and regeneration. Numerous studies have shown that a variety of signaling such as fibroblast growth factor (FGF), FGF receptor 2 (FGFR2), Notch, E-cadherin, bone morphogenetic protein (BMP), transforming growth factor-β (TGF-β), Activin, and Follistatin participate in regulation of proliferation and differentiation of DESCs [1, 3–7]. However, it still remains unknown whether the NF-κB signaling pathway participates in the regulation of DESCs.

In mammals, NF-κB family consists of five subunits, NF-κB1 (p50), NF-κB2 (p52), RelA (p65), RelB, and c-Rel, which can form homo- or hetero-dimeric complexes [8, 9]. In unstimulated cells, NF-κB is restricted to the cytoplasm in an inactive form by combining with inhibitors of ΚB (IκB). After various stimulations, the canonical NF-κB signaling pathway is activated. The IκB kinase (IKK) complex composed of two catalytic subunits, IKKα and IKKβ, and a regulatory subunit IKKγ, is activated and further brings about phosphorylation, ubiquitination, and proteasomal degradation of IκB. NF-κB dimers, predominantly the p50/p65 dimer, detach from IκB, permitting NF-κB dimers to translocate to the nucleus and regulate the target gene transcription [8]. NF-κB signaling pathway participated in many physiological and pathological processes such as cell proliferation, differentiation, apoptosis, inflammation, and immune response by regulating the expression of various genes [10, 11]. Moreover, the cross-talk between canonical NF-κB signaling pathway and many other signaling pathways such as Notch signaling, TGF-β/SMAD signaling, p53 signaling, and PI3-Kinase/AKT pathway has been widely studied in the regulation of physiological and pathological processes [12–15].

The canonical NF-κB signaling pathway plays a significant role in the biological functions of odontogenic mesenchymal stem cells including stem cells from apical papilla (SCAPs) and dental pulp stem cells (DPSCs) [16, 17]. Meanwhile, the canonical NF-κB signaling pathway can modulate odonto/osteogenesis of periodontal ligament stem cells (PDLSCs) and DPSCs in inflammatory microenvironments [16, 18, 19]. Numerous studies have showed that the NF-κB signaling pathway plays a pivotal role in odontogenesis. Ectodysplasin/NF-κB signaling is essential for tooth development. Defects in genes such as EctodysplasinA1 (EdaA1) (tumor necrosis factor [TNF] ligand; Tabby), Edar (TNF receptor; Downless), and Edaradd (death domain adaptor; Crinkled) lead to severely abnormal cusps and molar teeth in mice and hypohidrotic ectodermal dysplasia (HED) in human [20–25]. Furthermore, Ohazama et al. suggested that Ikkα was involved in cusp formation through the NF-κB pathway [26]. Mice overexpressing Ikkβ displayed supernumerary incisors, which suggested that excess NF-κB induced ectopic odontogenesis in embryonic incisor epithelium [27]. Therefore, the regulation of NF-κB signaling on DESCs needs us to investigate further.

In this study, we hypothesized that canonical NF-κB signaling participated in the regulation of DESC differentiation. To test this hypothesis, we used tumor necrosis factor-α (TNF-α), a best-studied activator of the canonical NF-κB pathway, and 4(2′-aminoethyl) amino-1,8-dimethylimidazo(1,2-a) quinoxaline (BMS-345541), a highly selective inhibitor of IKK, to activate and inhibit the NF-κB signaling pathway of DESCs, respectively [8, 28]. Furthermore, downregulation of SMAD7 using SMAD7 siRNA and activating of TGF-β signaling pathway using recombinant TGF-β were implemented in DESCs. In addition, we detected the changes of amelogenesis-related genes and proteins and TGF-β-SMAD signaling components.

## Materials and methods

### Cells isolation, culture, and identification

Sprague Dawley (SD) rats used in this experiment were commercially purchased from the experimental Animal Laboratory of Sichuan University. All experimental procedures were approved by the Ethics Committee of West China College of Stomatology, Sichuan University, Chengdu, China. SD rats on postnatal day (PN) 1 were sacrificed, and their mandibles were dissected immediately after sacrifice. Tissue from cervical loop regions was dissected from the mandibular incisors using the microdissection under aseptic conditions. The dissected tissue was incubated in 0.5% trypsin for 10 min at 37 °C. The samples were then cultured in epithelial cell medium (ScienCell, USA) consisting of basal medium, 2% fetal bovine serum (Hyclone, USA), 1% epithelial cell growth supplement, and 1% penicillin/streptomycin solution. When cells covered the flask, primary cells were incubated in 0.5% trypsin for 3–5 min at 37 °C to purify the epithelial cells. After two to three times of purification, almost all adherent cells were epithelial cells. All cells were cultured at 37 °C in a humid atmosphere with 5% CO2, and the medium was changed every other day.

DESCs were identified by using immunocytochemistry staining. Cells cultured in six-well plates (Becton Dickinson) were incubated with mouse anti-CK14 (1:200, ab49747, Abcam, Cambridge, MA, USA), mouse anti-vimentin (1:200, OMA1-06001, thermo), rabbit anti-integrin β1 (1:250, ab52971, Abcam, Cambridge, MA, USA), and rabbit anti-Sox2(1:200, ab97959, Abcam, Cambridge, MA, USA) overnight at 4 °C. Secondary antibodies fluorescein isothiocyanate-conjugated AffiniPure goat anti-mouse and goat anti-rabbit were applied for 1 h at 37 °C. The cell nuclei were stained with 40,6-diamidino-2-phenylindole (DAPI; Sigma-Aldrich) for 5 min at room temperature, and photos were obtained using a fluorescence microscope (Leica DMI 6000, Germany).

DESCs were also identified by using flow cytometry. The DESCs were collected and centrifuged at 1500 rpm for 5 min after trypsinization. Then, DESCs were fixed with 4% paraformaldehyde for 15 min at room temperature. 0.1% Triton X-100, membrane breaking agent, was used to treat DESCs for 15 min at room temperature. DESCs were incubated with antibodies for 1 h in the dark at room temperature after washing with flow buffer. The antibodies used in this experiment were mouse anti-CK14 (1:100, sc-58733 FITC, Santa Cruz Biotechnology, CA, USA), rabbit anti-Vimentin (1:100, ab185030, Abcam, Cambridge, MA, USA), rabbit anti-integrin β1 (1:100, ab225269, Abcam, Cambridge, MA, USA), and mouse anti-Sox2 (1:100, sc-365964 AF488, Santa Cruz Biotechnology, CA, USA). Then, DESCs were transferred to the flow tubes and detected by the flow cytometer (Attune NxT, USA).

### Cell proliferation assay

DESCs were seeded into 96-well plates (Becton Dickinson) at a cell density of 1 × 10^3^ cells/well for 24 h. Cells were treated with 0.01 ng/ml, 0.1 ng/ml, 1 ng/ml, 10 ng/ml, and 50 ng/ml TNF-α and different concentrations of BMS-345541 including 0.01 μmol/L, 0.1 μmol/L, 1 μmol/L, 10 μmol/L, and 50 μmol/L. After treatment, the cells were enumerated using a Cell-Counting kit-8 (CCK-8, Dojindo, Tokyo, Japan) according to the manufacturer’s instructions every day. Briefly, 100 μl serum-free epithelial cell medium with 10 μl reagent was added to each well and incubated at 37 °C for 1 h. The reaction was measured at 450 nm using a spectrophotometer (Thermo Scientific Varioskan Flash, Thermo Scientific).

### siRNA interference experiment

The non-silencing control siRNA and SMAD7 siRNA duplexes were synthesized from RiboBio [29]. The siRNA duplexes against SMAD7 (RiboBio, China) was exhibited as follows: 5ʹ-GGCTGGAGGTCATCTTCAA-3ʹ. Transfection of these siRNA duplexes was conducted in six-well plates using riboFECTTM CP Reagent (RiboBio, China) following the manufacturer’s manual. Gene-silencing effect was evaluated by quantitative real-time PCR (qRT-PCR).

### RNA extraction and qRT-PCR

Total RNA was extracted from DESCs with RNAisoTM Plus (TaKaRa Biotechnology, Tokyo, Japan). The complementary DNA synthesis was performed using a RevertAid First Strand cDNA Synthesis Kit (Thermo Fisher Scientific, Waltham, MA, USA) from the extracted RNA. qRT-PCR was performed via ABI7300 real-time PCR System (Applied Biosystems, Inc., USA) using the following cycling conditions: 95.0 °C for 30 s, 40 cycles of 95.0 °C for 5 s, and 60.0 °C for 30 s, and a melt curve cycle of 95.0 °C for 15 s, 60.0 °C for 1 min, and 95.0 °C for 15 s. Primers are listed in Table 1. We used glyceraldehyde 3-phosphate dehydrogenase (GAPDH) as the internal control, and the results were performed at least three times.

**Table 1:** Primer sequences

Gene	Primer forward (5^′^-3^′^)	Primer reverse(5^′^-3^′^)
AMBN	CTGCTCCTGTTCCTGTCCCTA	GCTTCCCAACTGTCTCATTGTC
MMP20	GCCTTGCTGTCCTTGTCAC	GAGGTGGTAGTTGCTCCTGAAG
KLK4	CCGAACTACAATGACCCTTCTT	TCAGATGCTACCGAGAGATTCA
SMAD7	CCTCCTTACTCCAGATACCC	ATTCGGACAA CAAGAGTCAG
GAPDH	TATGACTCTACCCACGGCAAG	TACTCAGCACCAGCATCACC

### Western blot and gray level analysis

The cultured DESCs were lysed using radioimmunoprecipitation buffer, and total protein was obtained by centrifuging at 12,000 rpm for 15 min at 4 °C. Then, we used BCA Protein Assay kit (Pierce Biotechnology, Rockford, IL, USA) to measure the protein concentration. The same mass protein of each sample was loaded on 12% sodium dodecyl sulfate-polyacrylamide gel, and then electroblotted it onto polyvinylidene difluoride membranes (Bio-Rad, Hercules, CA, USA). After blocking with blocking buffer, the membranes were incubated with primary antibodies overnight at 4 °C, and then incubated with horseradish peroxidase-conjugated secondary antibody. The primary antibodies used in this experiment were rabbit anti-NF-κB p65 (1:1000, ab7970, Abcam, Cambridge, MA, USA), rabbit anti-matrix metalloproteinase 20 (MMP20) (1:1000, ab76109, Abcam, Cambridge, MA, USA), rabbit anti-p-SMAD2/3 (1:1000, ab63399, Abcam, Cambridge, MA, USA), rabbit anti-SMAD4 (1:1000, ab40759, Abcam, Cambridge, MA, USA), rabbit anti-p-NF-κB p65 (1:1000, sc-101751, Santa Cruz Biotechnology, CA, USA), rabbit anti-ameloblastin (AMBN) (1:1000, sc-50534, Santa Cruz Biotechnology, CA, USA), rabbit anti-kallikrein 4 (KLK4) (1:1000, sc-20622, Santa Cruz Biotechnology, CA, USA), rabbit anti-SMAD2/3 (1:1000, sc-8332, Santa Cruz Biotechnology, CA, USA), rabbit anti-SMAD7 (1:1000, D160746, Sangon Biotech), and mouse anti-GAPDH (1:10000, 200306-7E4, Zen). Protein was visualized with Amersham ECL Select Western blotting detection reagent (GE) in accordance with the manufacturer’s protocol. In addition, quantitative of gray value of Western blotting strip by using software ImageJ. The statistical significance between means of two groups was assessed by *t* test using statistical software SPSS 16.0. *P* ≤ 0.05 was considered significant.

### Statistical analysis

All data were collected from three independent experiments and expressed as means ± standard deviation. The statistical significance between means of multiple groups was assessed by ANOVA and between means of two groups was assessed by *t* test using statistical software SPSS 16.0. *P* ≤ 0.05 was considered significant.

## Results

### Culture and identification of DESCs

**Fig. 1:**
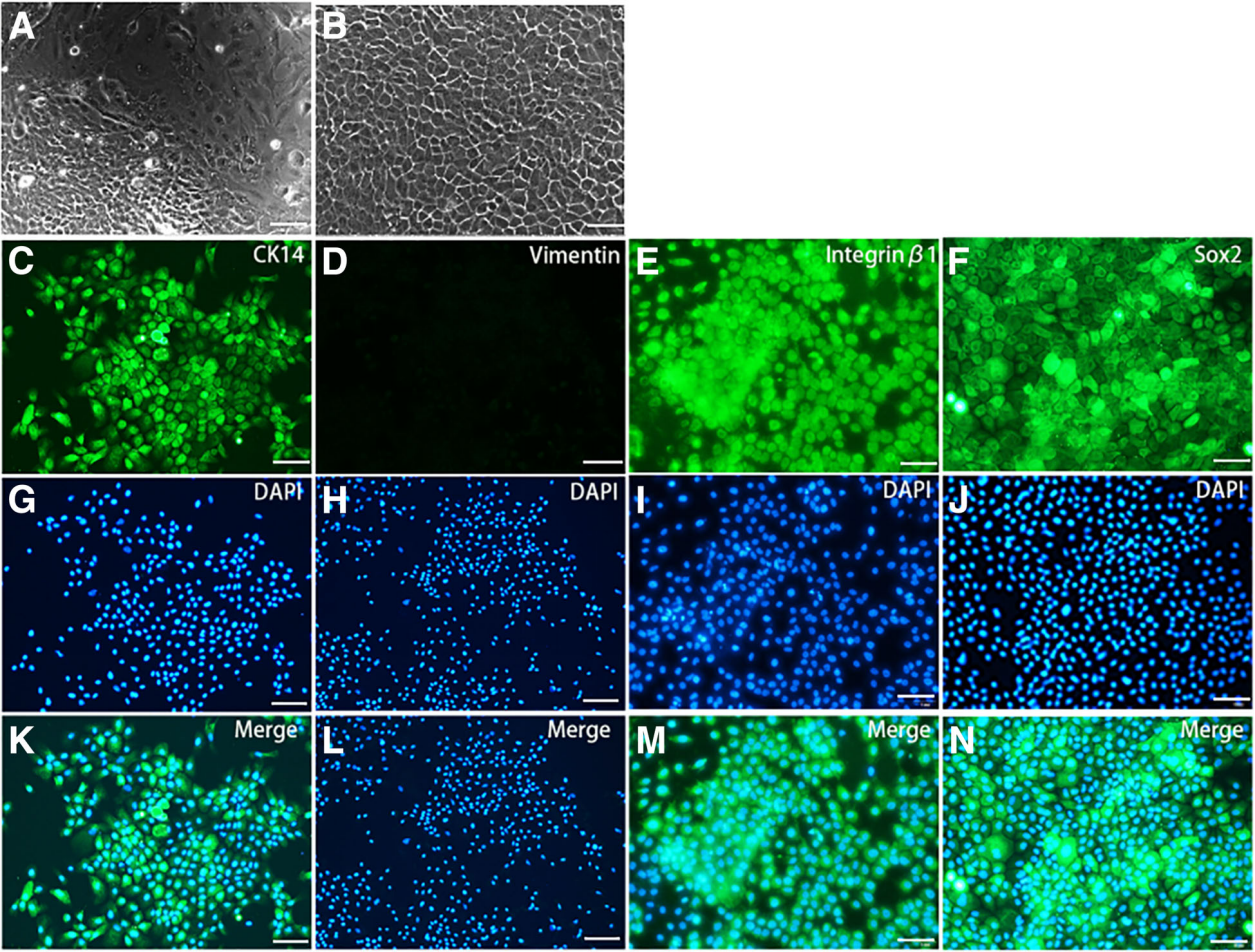
The culture and identification of DESCs. **a** The primary culture DESCs display a mixed form. **b** The purification DESCs had a polygonal shape and displayed a typical cobblestone morphology. **c**–**n** Immunofluorescence staining for CK14, vimentin, integrin-β1, and Sox2 in DESCs. **c**, **g**, **k** CK14 was positively expressed in DESCs. **d**, **h**, **l** Vimentin was negatively expressed in DESCs. **e**, **i**, **m** Integrin-β1 has strong expression in DESCs. **f**, **j**, **n** Sox2 was strong expression in DESCs. **a**–**n** Scale bar is 50 μm

After an attachment period of 24 h, primary cells climbed out from the tissue blocks. The primary cells presented a mixed form, which included epithelial stem cells exhibiting a polygonal shape and typical cobblestone morphology and mesenchymal stem cells displaying a fusiform shape (Fig. 1a). After repeated purification, almost all the cells were epithelial cells (Fig. 1b). Immunocytochemistry assay found that CK14, the gold standard marker of epithelial cells, was almost 100% positive (Fig. 1c, g, k), and integrin-β1 which was considered as a putative epidermal stem cell marker for characterizing epithelial stem cells strongly expressed in our purified epithelial cells [30, 31] (Fig. 1e, i, m). Sox2, the trustworthy and characterized dental epithelial stem cell marker, was strongly expressed in the epithelial cells (Fig. 1f, j, n). In addition, the mesenchymal cell marker vimentin showed negative staining (Fig. 1d, h, l). Consistent with the immunocytochemistry assay, flow cytometry analysis revealed that CK14, integrin-β1, and Sox2 were also strongly expressed in DESCs. In addition, the mesenchymal cell marker vimentin also showed negative staining (Additional file 1: Figure S1). These results indicated that the DESCs used in this experiment were a pure population.

### Activation and inhibition of canonical NF-κB signaling in DESCs

**Fig. 2:**
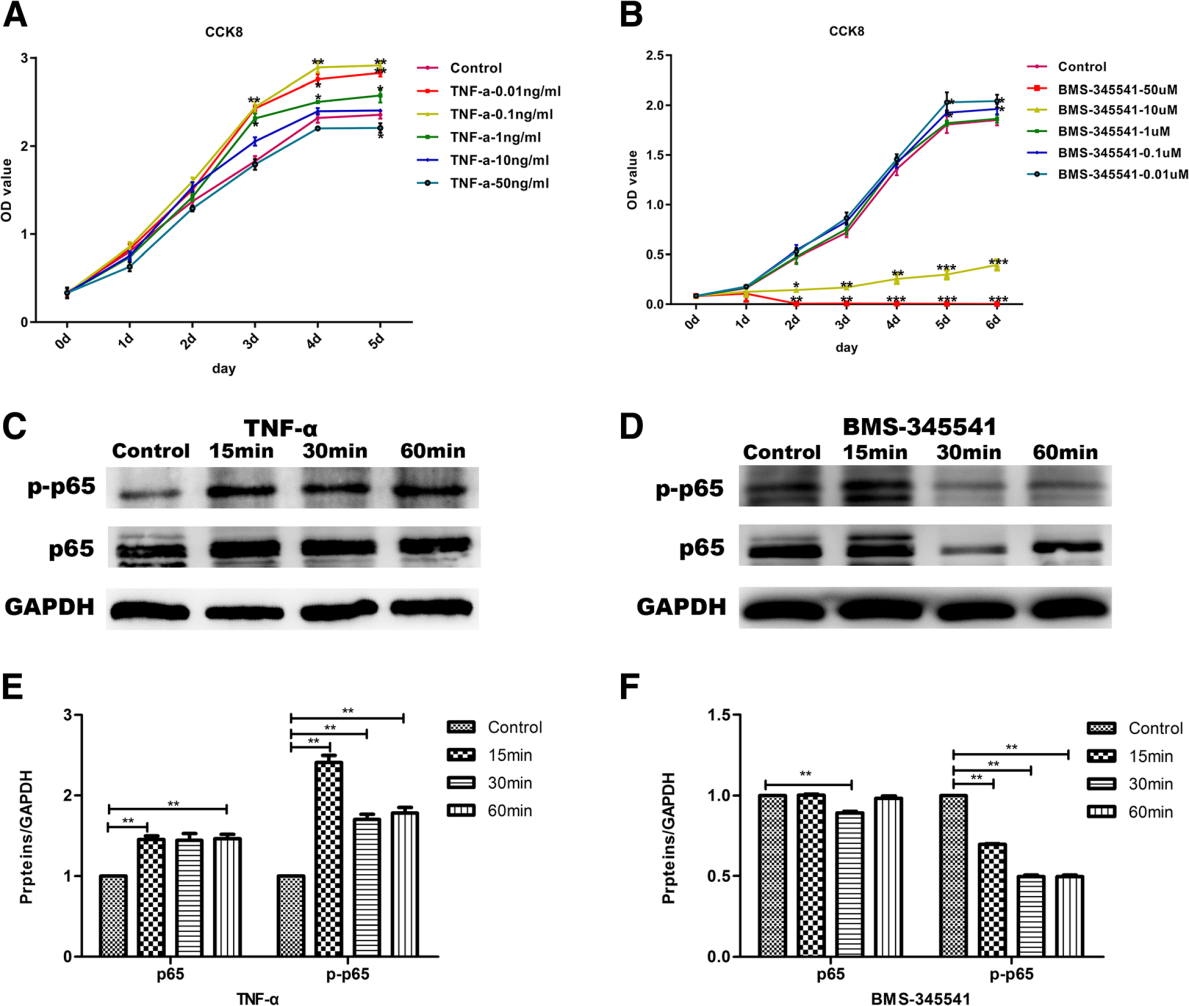
Activation and inhibition of NF-κB signaling pathway in the DESCs using TNFα and BMS-345541. **a**, **b** The effects of TNFα and BMS-345541 with five different concentrations on the proliferation of DESCs were analyzed by CCK8 assay. **c** The expression of p65 and p-p65 associated with canonical NF-κB signaling pathway were increased after treating with TNFα at different time points. **d** Western blot analyses revealed that the expression of p65 and p-p65 were downregulated with the treating of BMS-345541 at different time points. **e** Gray level analysis of protein expression of p65 and p-p65 in TNFα-treated DESCs. **f** Gray level analysis of protein expression of p65 and p-p65 in BMS-345541-treated DESCs. All values were presented as the means ± SD of triplicate experiments. *P < 0.05, **P < 0.01, and ***P < 0.001 (treatment group vs. control group)

In this study, we used CCK-8 to determine the optimum concentration of TNF-α and BMS-345541. The results showed that 50 ng/mL TNF-α efficiently inhibited the proliferation of DESCs. The proliferation of 10 ng/mL TNF-α group was similar to the control. In addition, TNF-α promoted the proliferation of DESCs at a relative low concentration (1 ng/mL, 0.1 ng/mL, and 0.01 ng/mL) (Fig. 2a). Meanwhile, low concentrations of BMS-345541 such as 0.01 μmol/L and 0.1 μmol/L promoted the proliferation of DESCs. One micromole per liter BMS-345541 had no obvious effect on cell proliferation, which displayed a similar proliferation rate to the control group. However, 10 μmol/L BMS-345541 inhibited the proliferation of DESCs, and 50 μmol/L BMS-345541 had a cellular cytotoxicity on DESCs (Fig. 2b). According to these results, we determined to use 10 ng/mL TNF-α and 1 μmol/L BMS-345541 to activate and inhibit the canonical NF-κB signaling pathway in the following experiments related to differentiation.

Next, we evaluated the effect of 10 ng/mL TNF-α and 1 μmol/L BMS-345541 on activation and inhibition of the canonical NF-κB signaling pathway in rat DESCs by using Western blot analysis. The results indicated that the expression of p-p65 was significantly increased with the treatment of 10 ng/mL TNF-α for 15 min (Fig. 2c, e). Meanwhile, the NF-κB signaling pathway was noticeably inhibited, as the results showed that p-p65 was significantly decreased in the 1 μmol/L BMS-345541-treated DESCs (Fig. 2d, f). These results indicated that the canonical NF-κB signaling pathway of DESCs was activated or inhibited by TNF-α and BMS-345541, respectively.

#### Canonical NF-κB signaling pathway participated in the regulation of cervical loop epithelial stem cell differentiation

**Fig. 3:**
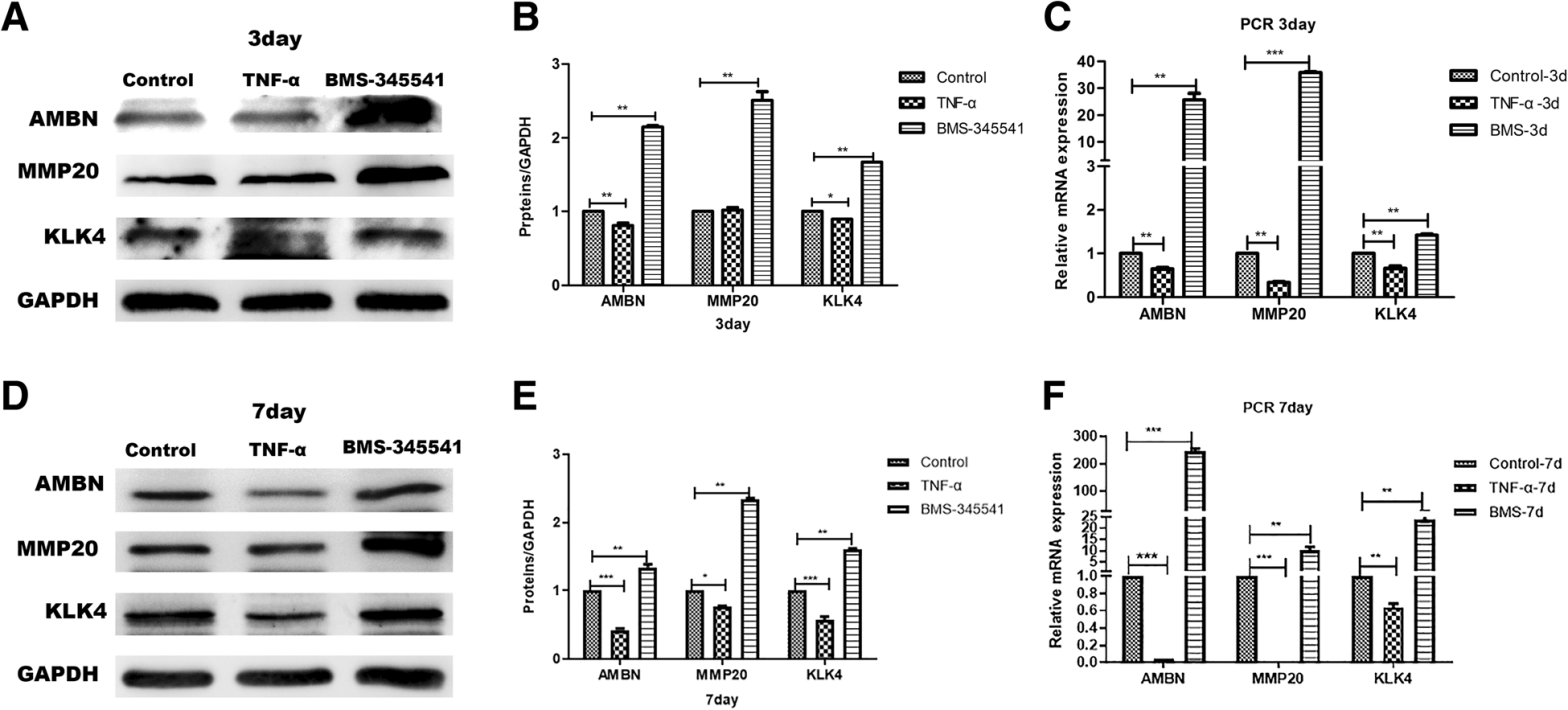
Amelogenesis-related protein and gene expressions were analyzed by Western blot and qRT-PCR tests in NF-κB-activated and NF-κB-inhibited DESCs. **a** Western blot analyses for the expressions of AMBN, MMP20, and KLK4 in different groups at day 3. GAPDH served as an internal control. **b** Gray level analysis of amelogenesis-related protein at day 3. **c** The gene expression of AMBN, MMP20, and KLK4 was measured by qRT-PCR test at day 3. **d** Western blot analyses for the expressions of amelogenesis-related protein in each group at day 7. GAPDH served as an internal control. **e** Gray level analysis of amelogenesis-related protein at day 7. **f** qRT-PCR analysis for amelogenesis-related genes in different groups at day 7. All values were presented as the means ± SD of triplicate experiments. *P < 0.05, **P < 0.01, and ***P < 0.001

We first investigated whether the canonical NF-κB signaling pathway regulated the differentiation of DESCs by using 10 ng/ml TNF-α or 1 μmol/L BMS-345541 to activate or inhibit the canonical NF-κB signaling pathway of DESCs, respectively. Western blot and gray level analysis revealed that protein expression levels of AMBN, MMP20, and KLK4 were faintly decreased in the TNF-α-treated group at day 3. Moreover, these proteins were increased in the BMS345541-treated group at day 3 (Fig. 3a, b). In addition, the expression levels of these amelogenesis-related proteins were specifically downregulated in the TNF-α-treated group and increased in the BMS345541-treated group at day 7 (Fig. 3d, e).

Next, we also assayed the change of gene expression by using real-time qRT-PCR. Consistent with the result of Western blot analysis, gene expression was slightly downregulated at day 3 and significantly suppressed at day 7 in the TNF-α-treated group. Furthermore, these genes were significantly upregulated in the BMS345541-treated group at both day 3 and day 7 (Fig. 3c, f). These results preliminarily showed that canonical NF-κB signaling pathway regulated the differentiation of cervical loop epithelial stem cells.

**Fig. 4:**
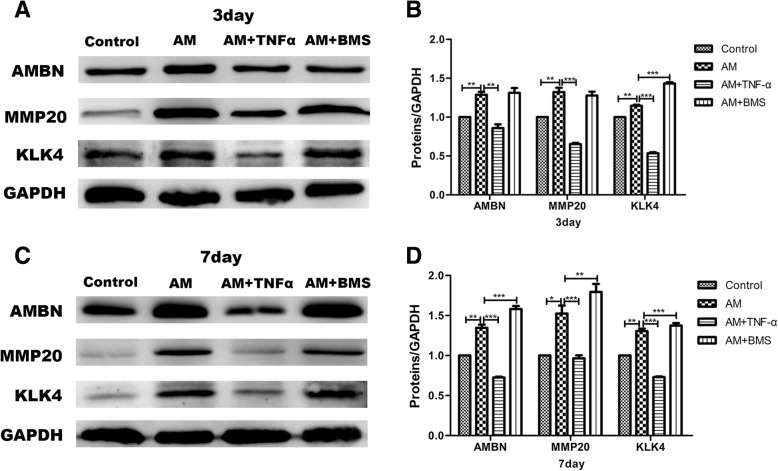
Analysis for amelogenesis of DESCs in control, amelogenesis-induction medium, amelogenesis-induction medium plus TNFα, and amelogenesis-induction medium plus BMS-345541 groups. **a**, **c** The protein expressions of AMBN, MMP20, and KLK4 were significantly elevated in the amelogenesis-induction group. Compared with the amelogenesis-induction group, treatment with TNF-α in the amelogenesis-induction medium had lower protein levels, while it had higher protein levels in amelogenesis-induction medium + BMS-345541 groups at day 3 and day 7. GAPDH served as an internal control. **b**, **d** Gray level analysis of amelogenesis-related protein in different groups at day 3 and day 7. All data were presented as the means ± SD of triplicate experiments. *P < 0.05, **P < 0.01, and ***P < 0.001. AM, amelogenesis-induction medium; BMS, BMS-345541

To further demonstrate the role of NF-κB signaling pathway on the DESC differentiation, we used amelogenesis induction in vitro and adding 10 ng/ml TNF-α or 1 μmol/L BMS-345541 to the amelogenesis-induction medium, respectively. After culturing for 3 days, Western blot and gray level analysis showed higher expression levels of AMBN, MMP20, and KLK4 in the amelogenesis-induction group compared with the control. Moreover, treatment with TNF-α in the amelogenesis-induction medium inhibited AMBN, MMP20, and KLK4 expression. Treatment with BMS-345541 in the amelogenesis-induction medium had no significant change on the protein expression level compared with the amelogenesis-induction group (Fig. 4a, b). Meanwhile, the results of 7 days were consistent with that of 3 days. The amelogenesis-related protein expression level was significantly elevated in the amelogenesis-induction group compared with the control group. The amelogenesis-related protein expression level of treatment with TNF-α in amelogenesis-induction medium was significantly decreased, whereas the amelogenesis-induction medium supplement with the BMS-345541 group was noticeably elevated compared with the amelogenesis-induction group (Fig. 4c, d). These data confirmed that activation of the NF-κB signaling pathway inhibited DESC differentiation; in contrast, inhibition of NF-κB signaling promoted DESC differentiation.

#### The cross-talk between TNF-α-induced canonical NF-κB signaling pathway and canonical TGF-β-SMAD signaling pathway during the DESC differentiation

**Fig. 5:**
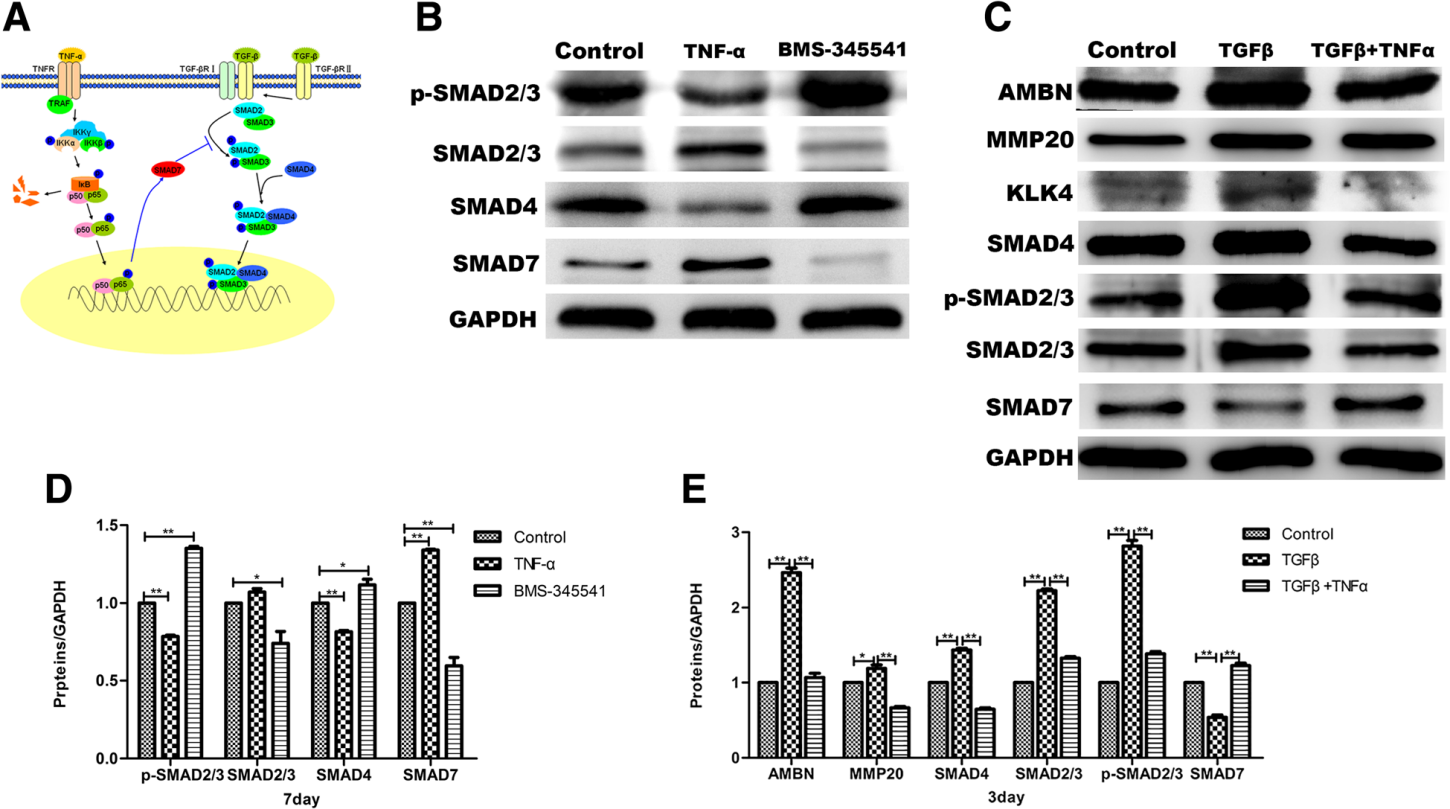
TNF-α-induced canonical NF-κB signaling inhibited the activation of canonical TGF-β-SMAD signaling pathway. **a** A novel model of the cross-talk between NF-κB and TGF-β-SMAD signaling pathway mediated by SMAD7 in DESC differentiation. Under the stimulation of TNF-α, canonical NF-κB signaling pathway is activated and IKK complex is recruited to TNF-α receptor-associated proteins (TRAF), where it is activated and further brings about phosphorylation, ubiquitination, and proteasomal degradation of IκB. These events allow translocation of prototypical p65/p50 dimer into the nucleus, which induces the SMAD7 expression and in turn suppresses the phosphorylation of SMAD2/3 to inhibit the TGF-β-SMAD signaling pathway. **b** The expressions of canonical TGF-β-SMAD signaling components (p-SMAD2/3, SMAD2/3, SMAD4) were downregulated in TNFα-treated DESCs, while increased in BMS-345541-treated DESCs. In contrast, the SMAD7 expression was increased in TNFα-treated DESCs, while downregulated in BMS-345541-treated DESCs by Western blot analyses. GAPDH served as an internal control. **c** DESCs were preincubated with the recombinant TGF-β prior to stimulation with TNFα (10 ng/ml), and expressions of amelogenesis-related proteins and canonical TGF-β signaling components were detected by Western blot analysis. **d** Gray level analysis of the TGF-β-SMAD signaling components and SMAD7 in **b**. **e** Gray level analysis of proteins in **c**. All values were presented as the means ± SD of triplicate experiments. *P < 0.05, **P < 0.01, and ***P < 0.001

Now, we have demonstrated that canonical NF-κB signaling indeed regulates the differentiation of rat DESCs. However, the specific signal transduction mechanism is still unknown. Previous studies have shown that canonical IKK-NF-κB signaling represses TGF-β-SMAD signaling through upregulating the expression of SMAD7. In addition, TGF-β signaling involved in the regulation of DESCs. So, we hypothesized that p65 upregulates SMAD7 expression and further suppresses TGF-β-SMAD signaling to regulate the differentiation of DESCs (Fig. 5a). To examine the hypothesis, we detected the expression of canonical TGF-β-SMAD signaling components: p-SMAD2/3, SMAD2/3, SMAD4, and the expression of SMAD7 by using Western blot analysis. The Western blot results and gray level analysis showed that treatment with TNF-α inhibited the phosphorylation of SMAD2/3 and the expression of SMAD4, but promoted the expression of SMAD7. Conversely, treatment with BMS-345541 promoted the phosphorylation of SMAD2/3 and the expression of SMAD4, but decreased the expression of SMAD7 (Fig. 5b, d).

To further test the hypothesis, recombinant TGF-β was used to activate the TGF-β pathway and SMAD7 siRNA was used to downregulate the expression of SMAD7 in the DESCs. In the presence of recombinant TGF-β, the expressions of amelogenesis-related proteins (AMBN, MMP20, KLK4) and TGF-β-SMAD signaling component (p-SMAD2/3, SMAD2/3, SMAD4) were significantly higher than the control group, while the expression of these proteins was downregulated in cells treated with recombinant TGF-β plus TNFα compared to cells exposed to recombinant TGF-β alone. In addition, the SMAD7 expression in these groups is opposite to that of the above proteins (Fig. 5c, e).

**Fig. 6:**
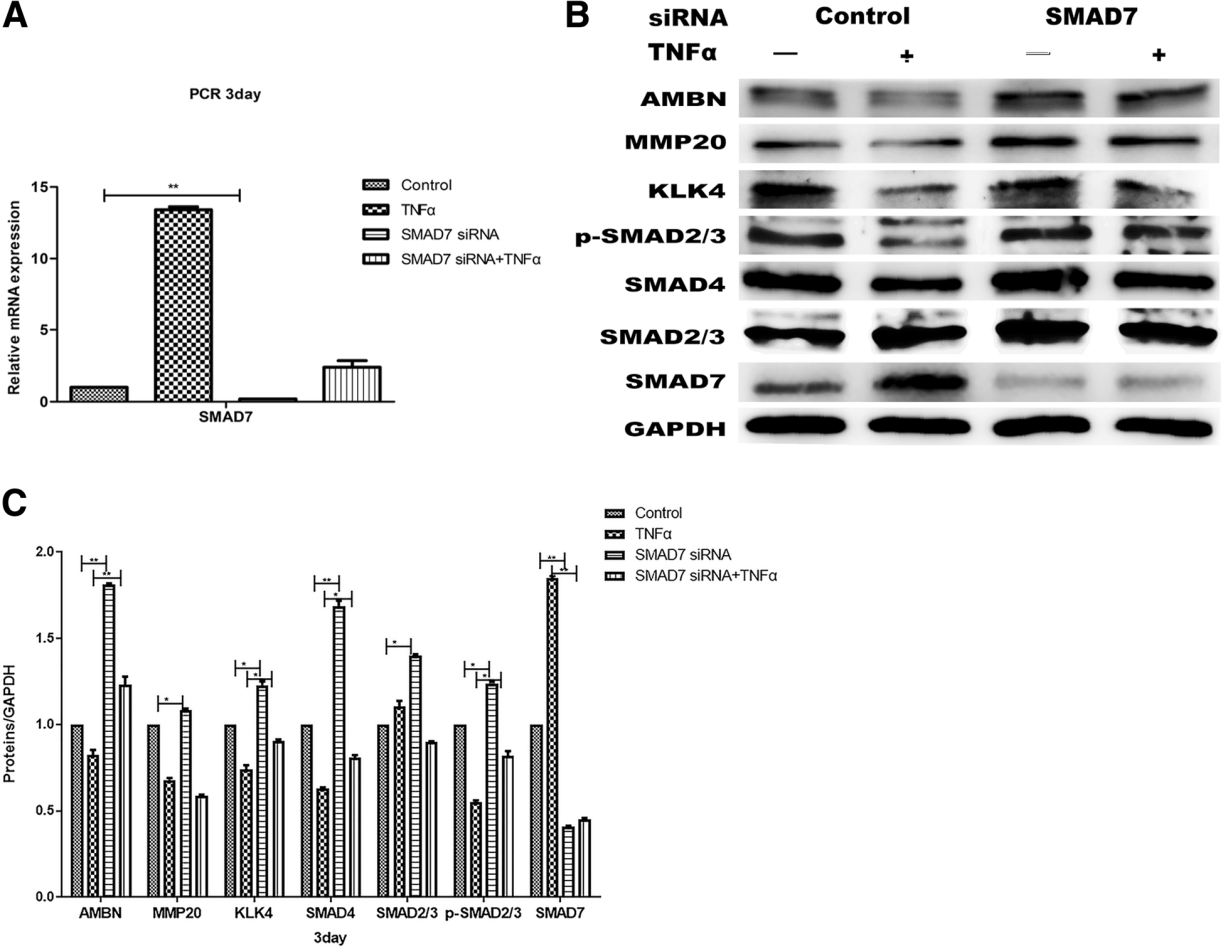
The cross-talk between NF-κB and canonical TGF-β signaling mediated by SMAD7 during the regulation of DESC differentiation. **a** Expression change of SMAD7 was estimated using qRT-PCR test. **b** DESCs were transfected with SMAD7-targeting and control non-targeting siRNA duplexes as indicated. After 2 h, cells were treated with TNFα (10 ng/ml) for 3 days. Expressions of amelogenesis-related proteins and canonical TGF-β signaling components were assessed by Western blot analysis. **c** Gray level analysis of proteins in **b**. All values were presented as the means ± SD of triplicate experiments. *P < 0.05, **P < 0.01, and ***P < 0.001

Furthermore, SMAD7 was downregulated using SMAD7 siRNA, thereby further exploring the DESC differentiation mechanism of cross-talk between NF-κB signaling pathway and TGF-β signaling pathway. We examined the expression change of SMAD7 gene using real-time qRT-PCR as shown in Fig. 6a. TNF-α increased levels of SMAD7 in control siRNA-transfected DESCs, but not in SMAD7 siRNA-transfected cells, indicating the successful blockage of SMAD7. Accordingly, TNF-α failed to decrease expressions of amelogenesis-related proteins (AMBN, MMP20, KLK4) and TGF-β-SMAD signaling component (p-SMAD2/3, SMAD2/3, SMAD4) in cells transfected with SMAD7 siRNA compared to cells transfected with control siRNA (Fig. 6b, c).

## Discussion

NF-κB signaling pathways involved in many physiological and pathological processes and played a key role in tooth development. The disorder of the NF-κB signaling pathway leads to abnormal tooth morphology such as the abnormal number of cusps and teeth and flattened cusps [21, 22, 25]. Furthermore, overexpression of NF-κB signaling in the embryonic incisor epithelium is able to form the supernumerary incisors [27]. Past studies showed that canonical NF-κB signaling indeed mediated the tooth development; however, it still remains unknown whether canonical NF-κB signaling pathway participates in the regulation of DESCs. Here, we demonstrated for the first time that canonical NF-κB signaling expressed in DESCs and involved in the regulation of DESC differentiation.

Canonical NF-κB signaling participates in the regulation of endothelial cell, bronchial epithelial cells, hepatic stellate cells, and intestinal epithelial cells for instance [32–36]. Previous studies have found the expression of molecules associated with the NF-κB signaling pathway in tooth epithelial during early tooth development [26]. Our previous experiments have shown that p65 and p-p65 were both detected in the cervical loop of rat incisors, which indicated that canonical NF-κB signaling indeed existed in the DESCs during postnatal tooth development. The stem cells from the cervical loop move towards the distal tip and differentiate into the ameloblast. AMBN is an enamel matrix protein which is secreted by ameloblasts and required to support rod formation and hydroxyapatite (HA) crystallization [37]. MMP20, the predominant secretory stage enzyme, is secreted by secretory stage ameloblasts, while KLK4, the predominant degradative enzyme, is secreted by transition and maturation stage ameloblasts [38]. The functions of MMP20 and KLK4 in the enamel formation are to clear proteins from the enamel matrix and to promote the orderly replacement of organic matrix with mineral [19]. Here, we demonstrated that the canonical NF-κB signaling pathway regulated the differentiation of DESCs; specifically, activation of the NF-κB signaling pathway inhibited the expression of amelogenesis-related proteins, whereas inhibition of the NF-κB signaling pathway promoted the expression of amelogenesis-related proteins. In addition, canonical NF-κB signaling also participated in the differentiation of dental MSCs. Inhibition of canonical NF-κB could recover the osteogenic differentiation potential of human PDLSCs from periodontitis patients (P-PDLSCs) [18]. Similarly, decreasing canonical NF-κB expression promotes odontoblastic differentiation and collagen formation of DPSCs in the presence of inflammatory cytokines [16]. Our conclusion was consistent with these studies.

TGF-β signaling plays a critical role in tissue homeostasis and cellular processes, including stem cell maintain and differentiation of DESCs [39–41]. NF-κB and TGF-β signaling pathways have a complex pattern of transmodulation. Here, we present a possible regulation mechanism in DESC differentiation, which TNF-α-induced canonical NF-κB/p65 signaling pathway suppresses canonical TGF-β signaling mediated by inducing SMAD7 expression to regulate the DESC differentiation (Fig. 6d). SMAD7, an inhibitory SMAD, competes with receptor-activated SMAD2/3 to inhibit canonical TGF-β signaling [42–45]. TNF-α-induced canonical NF-κB/p65 contributes to SMAD7 synthesis through activating the transcription of inhibitory SMAD7 [46]. In this study, we demonstrated that TNF-α-induced canonical NF-κB signaling upregulated the SMAD7 expression. Previous studies have shown that canonical NF-κB signaling suppresses TGF-β-induced physiological and pathological processes by upregulating the SMAD7 expression [13, 46, 47]. In fibroblasts, TNF-α-induced canonical NF-κB/p65 signaling induces SMAD7 to interfere with TGF-β type I receptor signaling, which decreases phosphorylation of substrate SMAD2/3 to suppress the canonical TGF-β signaling [46]. In addition, p65-NF-κB activation induces SMAD7 expression and represses TGF-β/SMAD-regulated gene PAI1 in head and neck cancers [13].

The continuously growing rodent incisor is identified as an excellent model to study stem cell function and regulation during tooth development [1]. In rodent incisor, the enamel-secreting ameloblasts exist exclusively in the labial portion, so the lingual surface is covered only by dentin and enamel-free [3, 7]. However, ectopic enamel deposition takes place on the lingual side of the incisor in mice which FGF signaling pathway is upregulated or mice with loss of function of sprouty genes [48]. The ablation of SMAD4 in the epithelium, a central intracellular mediator of the TGF-β signaling pathway, can prolong the maintenance of the cervical loop and crown development [49]. Wang et al. put forward an integrated gene regulatory network that involved in the regulation of stem cell proliferation in the cervical loop and asymmetric containing Activin, BMP, FGF, and Follistatin [6, 7, 48]. Previous studies have shown that NF-κB signaling pathway participated in the regulation of proliferation in several cell types, such as lymphocytes, mammary epithelial cells, and nasopharyngeal epithelial cells [50, 51]. However, whether canonical NF-κB signaling pathway regulates the proliferation and asymmetry of the cervical loop needs further study.

## Conclusion

DESCs, the only cells that secrete enamel, are important seed cells in tooth regeneration research. Therefore, our study mainly explored the differentiation regulation mechanism of the DESCs residing in the cervical loop of rat incisors. Our study has demonstrated that activation of the canonical NF-κB signaling pathway could inhibit canonical TGF-β-SMAD signaling pathway through upregulating SMAD7 expression and thereby inhibiting the differentiation of DESCs. In addition, SMAD7 was a key node factor in the cross-talk of the canonical NF-κB signaling pathway and canonical TGF-β-SMAD signaling pathway, which provided theoretical support for signal transduction factors in tooth regeneration.

## Additional file

Additional file 1: **Figure S1.** The identification of DESCs by using flow cytometry**.** Flow cytometry for CK14, vimentin, integrin-β1, and Sox2 in the purification DESCs. CK14, integrin-β1, and Sox2 were strongly expressed in DESCs. In addition, vimentin showed negative expression in DESCs. (TIF 508 kb)

## Abbreviations

AMBN: Ameloblastin
BMP: Bone morphogenetic protein
CCK-8: Cell-Counting kit-8
DAPI: 40,6-Diamidino-2-phenylindole
DESCs: Dental epithelial stem cells
DPSCs: Dental pulp stem cells
EdaA1: EctodysplasinA1
FGF: Fibroblast growth factor
FGFR2: FGF receptor 2
GAPDH: Glyceraldehyde 3-phosphate dehydrogenase
HED: Hypohidrotic ectodermal dysplasia
IKK: IκB kinase
IκB: Inhibitors of ΚB
KLK4: Kallikrein 4
MMP20: Metalloproteinase 20
PDLSCs: Periodontal ligament stem cells
PN: Postnatal day
qRT-PCR: Quantitative real-time PCR
SCAPs: Stem cells from apical papilla
SD: Sprague Dawley
TGF-β: Transforming growth factor-β
TNF: Tumor necrosis factor
