# Supplementary figures and images for: Effect of canonical NF-κB signaling pathway on the differentiation of rat dental epithelial stem cells
Source: Stem Cell Res Ther. 2019 May 20;10:139. doi: 10.1186/s13287-019-1252-7 (PMC6528379; doi:10.1186/s13287-019-1252-7)

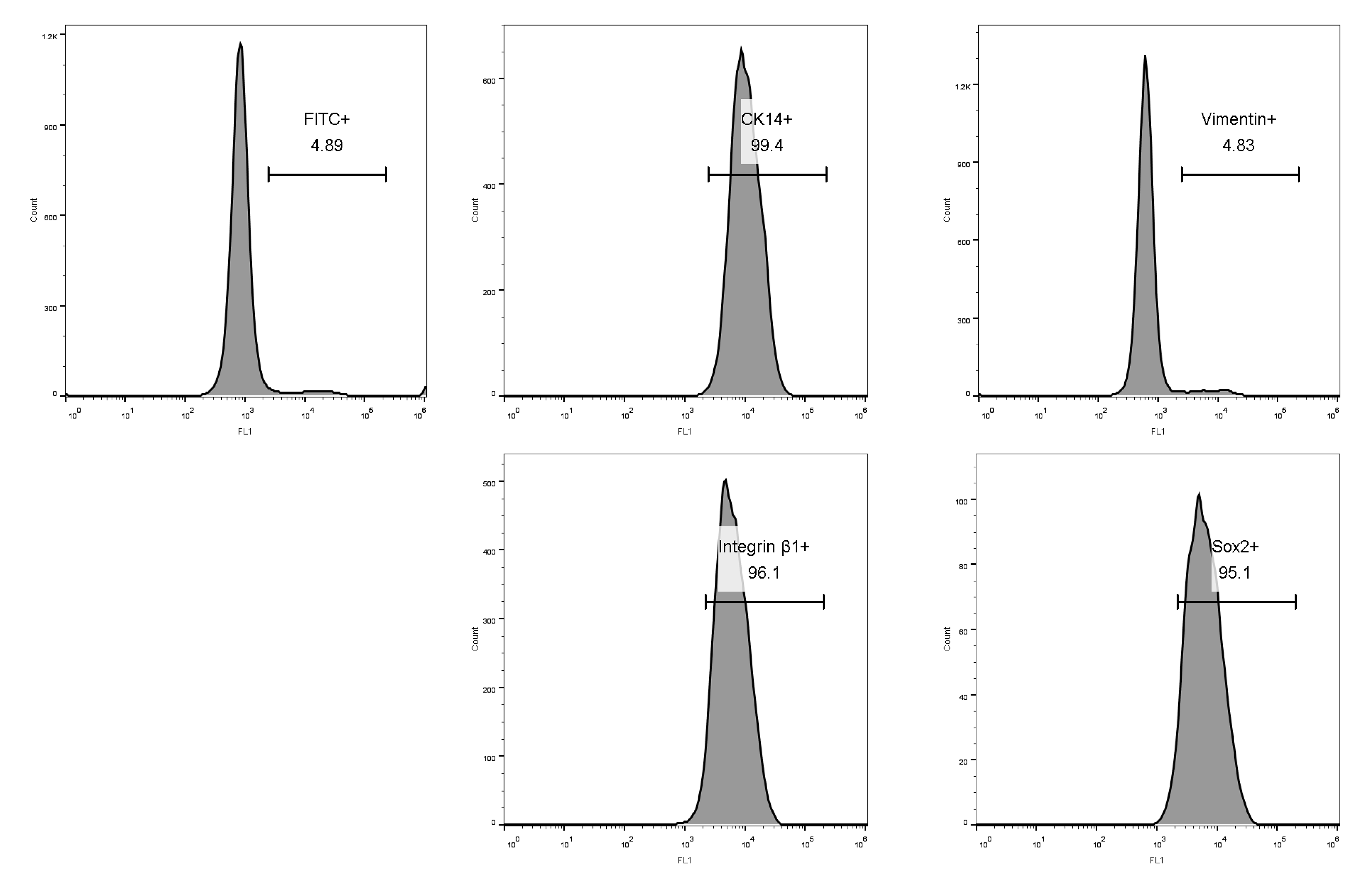

Supplement: Supplementary file 1 — Figure S1. The identification of DESCs by using flow cytometry. Flow cytometry for CK14, vimentin, integrin-β1, and Sox2 in the purification DESCs. CK14, integrin-β1, and Sox2 were strongly expressed in DESCs. In addition, vimentin showed negative expression in DESCs. (TIF 508 kb) [file 13287_2019_1252_MOESM1_ESM.tif]
